# Sedation and analgesia practices at Italian neonatal intensive care units: results from the EUROPAIN study

**DOI:** 10.1186/s13052-017-0343-2

**Published:** 2017-03-07

**Authors:** Paola Lago, Anna Chiara Frigo, Eugenio Baraldi, Roberta Pozzato, Emilie Courtois, Jérôme Rambaud, Kanwaljeet J. S. Anand, Ricardo Carbajal

**Affiliations:** 1 Neonatal Intensive Care Unit, Women’s and Children’s Health Department, Azienda Ospedaliera-University of Padova, Via Giustiniani 3, Padua, 35128 Italy; 2 Department of Cardiac, Thoracic and Vascular Sciences, University of Padua, Padua, Italy; 3 Hôpital Amand Trousseau, Service des Urgences Pédiatrique, Paris, France; 4 Hôpital Amand Trousseau, Service de Réanimation Pédiatrique et Néonatale, Paris, France; 5 Departments of Pediatrics, Anesthesiology, Perioperative and Pain Medicine, Stanford University School of Medicine, Stanford, CA USA; 6 Université Pierre at Marie Curie, Faculté de Médecine, Paris, France

**Keywords:** Pain, Newborn and preterm, Analgesic, Sedative, Opioids, Pain assessment

## Abstract

**Background:**

We aimed to examine current bedside analgesia/sedation (A/S) and pain assessment (PA) practices in Italian neonatal intensive care units (NICUs) in relation to the findings of an epidemiological European study and recently-introduced national guidelines.

**Methods:**

We analyzed the Italian data from the EUROPAIN (EUROpean-Pain-Audit-In-Neonates) prospective observational study on A/S practices that involved 6680 newborns admitted to tertiary-level NICUs in 18 European countries. Demographics, type of assisted ventilation, type and mode of A/S administration and PA were analyzed. Multivariate linear regression models were used to identify factors predicting A/S and PA practices.

**Results:**

From October 1^st^, 2012 to June 30^th^, 2013, thirty Italian NICUs gathered data on 422 newborn: 131 on invasive ventilation (IV); 150 on noninvasive ventilation (NIV); and 141 on spontaneous ventilation (SV). A/S was documented for 35.3% of all infants admitted (86.3% IV; 17.3% NIV; 7.1% SV [*p* = 0.0001]), and varied considerably between NICUs (as reported in other European countries). Strong analgesics were used in 32.5% of cases, sedatives in 10.2%, mild analgesics in 3.8%. Fentanyl was used in 78.6% of cases, morphine in 8.4%, neuromuscular blockers in 5.3%, midazolam in 22.1%. The performance of PA was documented in 67.5% of all newborn (85.5% IV; 67.3% NIV; 51.1% SV [*p* = 0.001]). Illness severity, type of ventilation, bedside PA, and number of NICU beds were all factors associated with A/S use on multivariate analysis, while gestational age ≤ 32 weeks, and type of ventilation and presence of a pain team were associated with PA.

**Conclusions:**

We documented a generally widespread, but still highly variable use of A/S and PA at Italian NICUs, despite the diffusion of national guidelines. There is an urgent need to improve routine PA to enable customized pain and stress control (and prevention) in all infants.

**Trial registration:**

Clinical Trials.gov # NCT01694745.

**Electronic supplementary material:**

The online version of this article (doi:10.1186/s13052-017-0343-2) contains supplementary material, which is available to authorized users.

## Background

The use of analgesia and sedation (A/S) in newborns has increased largely in the last 25 years since it was demonstrated that failure to administer analgesics during neonatal anesthesia increased infants’ stress hormone response, time to recovery, and mortality rates [1, 2].

Researchers also showed that the exposure of term and preterm newborns to uncontrolled and repetitive pain is most common in intensive care units [3] and may affect the infants’ pain perception in later infancy [4], and impair their neurodevelopmental outcome in terms of cognition [5], motor function [6] and brain development [7, 8].

Further studies demonstrated the efficacy of A/S during mechanical ventilation in reducing pain scores, but they were unable to demonstrate any impact on neurodevelopmental outcome or survival [9]. Some concerns have also been raised concerning the potential toxicity of strong analgesics, sedatives and anesthetic drugs in the neonatal period, prompting a more judicious use of these drugs at this crucial time for brain development [10, 11]. There have been reports of analgesic therapy prolonging the need for mechanical ventilation, delaying feeding [12], and possibly contributing to other sequelae, including impaired brain growth, altered eye-hand coordination, and weak short-term memory [13, 14].

Since the early 2000s, scientific societies and consensuses on pain control and prevention in the newborn have recommended using pharmacological and non-pharmacological interventions for all painful and stressful neonatal procedures, and routine pain assessments (PA) to enable analgesic intervention to be customized appropriately [15–17]. How much these best practices have been implemented remains unclear, however. In 2005 and 2010, we investigated this issue at Italian NICUs. Despite a widespread (albeit variable) use of A/S, we identified a very limited routine use of PA, and concluded that there remained an urgent need to implement routine PA in all newborn infants to better customize A/S in clinical practice [18–20].

The EUROPAIN survey is the first study, to our knowledge, to have prospectively recorded round-the-clock bedside A/S and PA practices for all NICU admissions over a defined period of time, generating a precise picture of these practices [21].

We analyzed, in detail, the Italian data obtained for the EUROPAIN survey to document and compare current A/S practices at Italian NICUs vis-à-vis their counterparts elsewhere in Europe and the latest national guidelines [22].

## Methods

### Study design and participants

The EUROPAIN (EUROpean-Pain-Audit-In-Neonates) survey was a prospective observational study on A/S management in NICU patients in 18 European countries. Details of the study are available at http://www.europainsurvey.eu/. Principal Investigators were appointed in each country to coordinate the study and contact all eligible tertiary-level NICUs (responsible for the care of critically-ill newborn of all gestational ages, that manage the full period of invasive ventilation). Coordinators of the nursing staff and physicians at each unit provided their general statistics and existing A/S protocols for the newborn. The national Principal Investigator also provided details of any national guidelines for treating or preventing neonatal pain.

### Data collection

All neonates up to 44 weeks post-conceptional age newly admitted to eligible Italian NICUs during the enrollment period were considered. Data were collected prospectively on each infant (demographics; modes of ventilation; use of continuous or intermittent/bolus sedation, analgesics or neuroblockers; drug withdrawal and pain assessment using any validated pain scale) for the first 28 days in hospital, or until death, discharge, or transfer to another hospital. Neonates were classified as belonging to the A/S group if they received at least one dose of such medication; the duration of A/S infusions or number of boluses were also recorded. As for PA, the NICUs were asked to specify what tools were used and the number of daily pain assessments. Neonates were deemed as assessed if at least one pain assessment was performed. Units recruited patients over a one-month period and data were collected on standardized paper questionnaires, then entered in online questionnaires. Each unit also kept a logbook of all neonates admitted during the study period. A centralized team in Paris monitored the completeness and relevance of the data entered in the study database, as reported in an earlier publication [21]. After obtaining the approval of the regulatory bodies for the Protection of Human Subjects, Data Protection, and Health Research Data Management in France, the study was also approved by the local ethical committees of the participating hospitals. Written informed consent was obtained from the parents of the infants involved.

The study was registered at ClinicalTrials.gov (#NCT01694745).

### Statistical analyses

The study population’s clinical characteristics were described in terms of numbers and percentages for categorical variables, medians with interquartile ranges for quantitative variables since not normally distributed (normality evaluated with Kolmogorov-Smirnov test). The distributions of patients’ clinical characteristics by A/S practices implemented were compared with the chi-square or Fisher’s exact test for categorical variables, and with Kruskal-Wallis rank sum test for quantitative variables because their distribution was not normal. Type and modality of A/S were compared between age groups (≤32 / >32) with the \chi-square or Fisher’s exact test. Predictors of A/S use and of PA were identified by means of a univariate hierarchical logistic regression model (SAS PROC GLIMMIX). The hierarchical model was adopted to take into account patient clustering at different units, considering the NICU as a random factor. We used model-building methods to obtain the best fit and the most parsimonious model. Laplace’s method was used to test the model’s fit by examining the change in the -2LL between models with the chi-square difference test. The variables identified as potential predictors in the univariate analyses to consider in the multivariate model were: gestational age (≤32 / >32 weeks), sex, severity of illness (Clinical Risk Index for Babies [CRIB] score, validated for all NICU admissions) [22], age on admission (≤6 / >6 h), 5-min Apgar score (<5 / ≥5), type of respiratory support, use of pain scales (for A/S use), presence of a pain specialist (physician or nurse) and pain team, presence of local written guidelines for pain (assessment and control), and characteristics of the NICUs in terms of numbers of beds (<15/≥15) and yearly admissions.

The results of the regression analyses are presented as odds ratios (OR) with two-sided 95% confidence intervals (CI).

Multicollinearity was checked with Variance Inflation Factor (VIF) and it was slightly above 1 indicating that multicollinearity was not present.

The statistical analyses were conducted with the SAS statistical package for Windows, release 9.4 (SAS Institute Inc., Cary, NC, USA).

## Results

### Study population

**Fig. 1:**
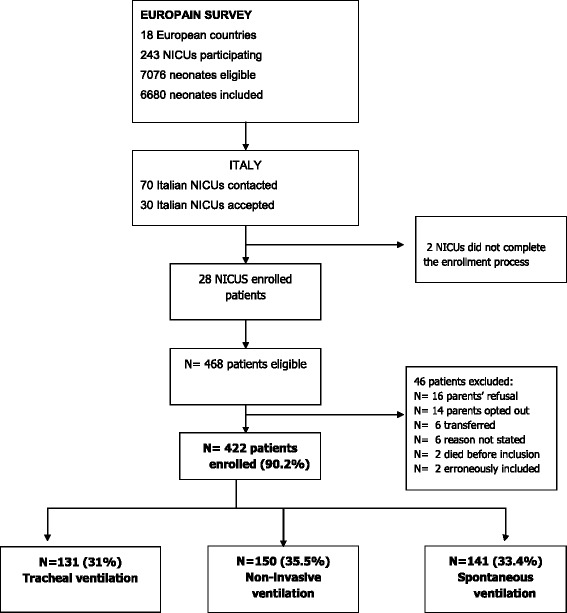
Study flowchart

From October 1^st^, 2012 to June 30^th^, 2013, 70 tertiary-level Italian NICUs meeting the above-defined entry criteria were invited to participate in the survey. Thirty (42.8%) accepted to join the study, but only 28 completed the data submission process. Of the 468 neonates enrolled, 46 were excluded, leaving a final sample of 422 newborns. Based on the highest level of respiratory support neonates received during their NICU stay, they were divided into three groups: 131 on invasive ventilation (IV); 150 on noninvasive ventilation (NIV); and 141 on spontaneous ventilation (SV). Figure 1 shows the trial flowchart.

Table 1 shows the study population’s demographics and clinical characteristics.

**Table 1:** Demographics of the study population

	Total (*n* = 422)	Invasive ventilation (*n* = 131)	Noninvasive ventilation (*n* = 150)	Spontaneous ventilation (*n* = 141)	*p*
Gestational age (weeks) median (IQR)	34.0 (31.0–37.0)	31.0 (27.0–36.0)	33.0 (31.0–35.0)	35.0 (34.0–38.0)	<.0001
Gestational age (weeks)					<.0001
24–29 N (%)	86 (20.4)	59 (45.0)	25 (16.7)	2 (1.4)	
30–32 N (%)	76 (18.0)	19 (14.5)	45 (30.0)	12 (8.5)	
33–36 N (%)	150 (35.6)	22 (16.8)	57 (38.0)	71 (50.4)	
37–42 N (%)	110 (26.1)	31 (23.7)	23 (15.3)	56 (39.7)	
Gestational age (weeks) ≤32 N (%)	162 (38.4)	78 (59.5)	70 (46.7)	14 (9.9)	< .0001
Birth weight (g) median (IQR)	1921 (1368–2739)	1600 (896–2550)	1878 (1367–2430)	2295 (1835–3130)	<.0001
Sex male N (%)	226 (53.6)	82 (62.6)	77 (51.3)	67 (47.5)	0.0356
Born in same hospital as NICU, n (%)	345 (81.8)	93 (71.0)	133 (88.7)	119 (84.4)	0.0004
Type of delivery Caesarean, n(%)	300 (71.1)	96 (73.3)	119 (79.3)	85 (60.3)	0.0013
Age at admission (h) median (IQR)	0.4 (0–3.3)	0.5 (0–3.2)	0.2 (0–0.6)	0.5 (0–19.1)	0.0017
CRIB score median (IQR)	0 (0–1)	2 (1–5)	0 (0–1)	0 (0–1)	<.0001
APGAR score at 5 min Median (IQR)	9 (8–10)	7 (6–9)	8 (8–9)	10 (9–10)	<.0001
Intubated on admission, n (%)	79 (18.7)	79 (60.3)	NA	NA	NA
Duration of IV (hours) median (IQR)	NA	71.7 (16.8–157.0)	NA	NA	NA
Duration of NIV (hours) median (IQR)	NA	*N* = 101 120 (33.0–262.8)	*N* = 150 36.1 (16.9–113.2)	NA	NA
Status at discharge Dead, n (%)	14 (3.3)	12 (9.2)	1 (0.7)	1 (0.7)	<.0001
Days of participation in trial median (IQR)	15 (7–28)	28 (13–28)	17 (8–28)	9 (5–15)	0.0001
Days of hospitalization median (IQR)	15 (7–28)	25 (13–47)	17.0 (8–28)	8.5 (5–15)	<.0001

Values were missing for some variables. *NA* Not applicable, *CRIB* Clinical Risk Index for Babies, *IV* invasive ventilation, *NIV* noninvasive ventilation, *SV* spontaneous ventilation

Most of the NICUs had local guidelines for controlling procedural and prolonged pain (*n* = 23 [82.1%]), for preventing and treating neonatal abstinence syndrome (*n* = 20 [71.4%]), and for pain monitoring (*n* = 23 [78.6%]); futhermore 82.1% of the units reported performing routine pain assessments. There was a doctor (*n* = 23 [82.1%]) and/or a nurse (*n* = 15 [71.4%]) responsible for pain treatment at the NICU, while pain teams were less common (*n* = 15 [53.6%]). Parents had unlimited access to the unit 24 h a day in 10 [36%] NICUs.

### Use of analgesia and sedation

The use of A/S varied greatly among NICUs, ranging from 5.3–100% of all NICU admissions (*p* < .0001), and from 50%–100% in infants on invasive ventilation (*p* = 0.0809).

We separately quantified the use of strong analgesics (morphine, fentanyl and ketamine), sedatives (midazolam, propofol), mild analgesics (paracetamol and local anesthetics) in the three differently ventilated groups, and the use of muscle relaxants only for the IV group, as shown in Table 2.

**Table 2:** Use of analgesia and sedation by type of ventilation

	Total (*N* = 422) n (%)	Invasive ventilation (*N* = 131) n (%)	Noninvasive ventilation (*N* = 150) N (%)	Spontaneous ventilation (*N* = 141) n (%)	*p*
Use of analgesia and/or sedation	149 (35.3)	113 (86.3)	26 (17.3)	10 (7.1)	<.0001
Method of administration:					
Bolus only	54 (36.2)	33 (29.2)	18 (69.2)	3 (30)	0.0035
Continuous only	34 (22.8)	28 (24.8)	4 (15.4)	2 (20)	
Continuous and bolus	61 (40.9)	52 (46.0)	4 (15.4)	5 (50)	
Strong analgesics^a^	137 (32.5)	109 (83.2)	21 (14.0)	7.0 (5.0)	<0.0001
Morphine	11 (2.6)	11 (8.4)	0 (0.0)	0 (0.0)	<0.0001
Fentanyl	131 (31.0)	103 (78.6)	21 (16.0)	7 (5.3)	<0.0001
Ketamine	2 (0.2)	2 (1.5)	0 (0.0)	0 (0.0)	0.0959
Sedatives^a^	45 (10.7)	35 (26.7)	5 (3.3)	5 (3.6)	<0.0001
Propofol	5 (1.2)	4 (3.1)	1 (0.7)	0 (0.0)	0.0434
Midazolam	38 (9.0)	29 (22.1)	4 (2.7)	5 (3.5)	<0.0001
Mild analgesics^a^	16 (3.8)	9 (6.9)	4 (2.7)	3 (2.1)	0.0823
Paracetamol	14 (3.3)	8 (6.1)	3 (2.0)	3 (2.1)	0.1369
Local anesthetics	2 (0.5)	1 (0.8)	1 (0.7)	0 (0.0)	0.7619
Neuromuscolar blockers	7 (1.7)	7 (5.3)	NA	NA	NA
Drug withdrawal	4 (2.8)	4 (3.6)	00 (0.0)	00 (0.0)	1.000
Pain assessment	285 (67.5)	112 (85.5)	101 (67.3)	72 (51.1)	<0.0001

^a^ only the main medications are described in detail

In the IV group, most infants (70.8%) were given continuous or continuous A/S plus boluses, while this was true of only 30.8% of infants in the NIV group (69.2% were given boluses of A/S).

In IV infants, fentanyl infusions lasted a mean ± SD of 184.5 ± 193.2 h, morphine was given for 95.9 ± 59.6 h, and midazolam for 117.2 ± 111.0 h. The use of neuromuscolar blockers was limited to 7 (1.7%) patients, with a mean infusion duration of 72.6 ± 85.4 h.

We documented much the same use of A/S at Italian NICUs (35.4%) as elsewhere in Europe (34.3%), be it for all admissions or by type of ventilation (IV 86.3% vs 81.2, NIV 17.3% vs 17.8%, and SV 7.1% vs 9.4% in Italy and other European countries, respectively). Significant differences in the mode of A/S administration emerged, however, with Italian NICU’s making less use of continuous infusions (CI) plus boluses in infants on IV (CI + Bolus: *n* = 948 [58.1%], ITA *n* = 52 [46,0%], only CI: EU *n* = 266 [16,3%], ITA *n* = 28 [24,8%], only bolus: EU *n* = 419 [25.7%], ITA *n* = 33 [29,2%], *p* = 0.0212), and of intermittent boluses alone in those on NIV (CI + bolus: EU *n* = 10 [4,2%], ITA *n* = 4 [15,4%], only CI: EU *n* = 1 [0,4%], ITA *n* = 4 [15,4%], only bolus EU *n* = 229 [95,4%], ITA *n* = 18 [69,2%], *p* < 0.0001) and SV (CI + bolus: EU *n* = 29 [10,7%], ITA *n* = 5 [50,0%], only CI: EU *n* = 8 [2,9%], ITA *n* = 2 [20,0%], only bolus EU *n* = 235 [86,4%], ITA *n* = 3 [30,0%], *p* < 0.0001) than in the rest of Europe.

**Fig. 2:**
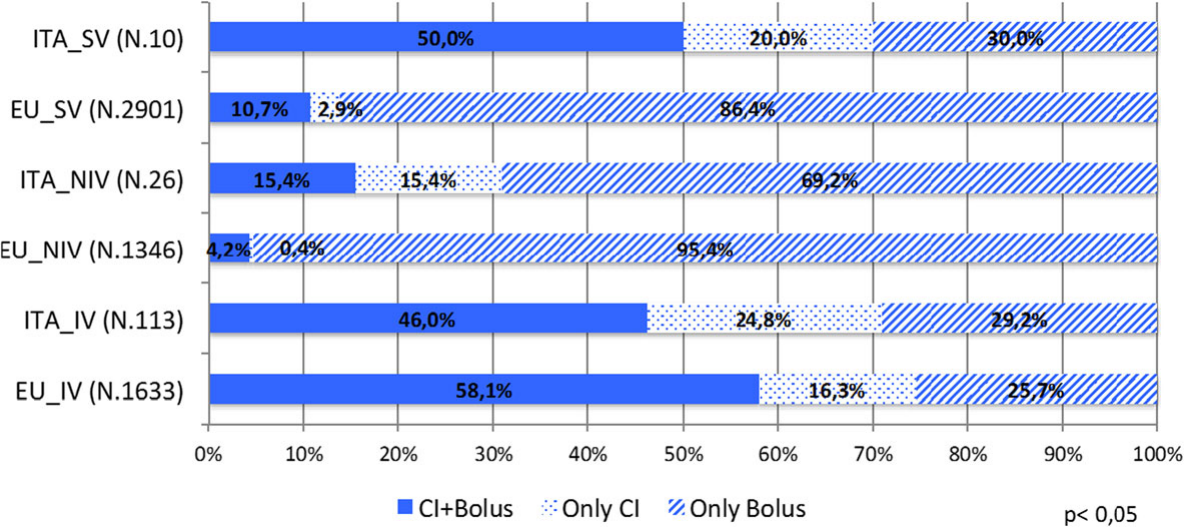
Analgesia and sedation in Italy and in all participating countries in Europe by type of ventilation. EU include: Austria, Belgium, Cyprus, Estonia, Finland, France, Germany, Greece, Lithuania, Malta, Netherlands, Norway, Poland, Portugal, Spain, Sweden, United Kingdom. Chi-square test *p* < 0.001

Figure 2 shows A/S practices in Italy versus elsewhere in Europe, by mode of administration.

### Use of analgesia and sedation by gestational age

The demographic characteristics of the very low birth weight infants born at less than 33 weeks of gestation are given in a supplemental table (Additional file 1: Table S1).

Data on the type of A/S and their mode of administration were also analyzed by gestational age group (Table 3).

**Table 3:** Type of analgesia and sedation, and their mode of administration by gestational age group

	Total *n* = 422		24–32 GA *n* = 162		33–42 GA *n* = 260		*P*
	N.	%	N.	%	N.	%	
Use of analgesia and/or sedation	149	35.3	87	53.7	62	23.8	0.001
Mode of A/S administration							
Bolus only	54	36.2	37	42.5	17	27.4	0.1671
Continuous only	34	22.8	18	20.7	16	25.8	
Bolus + continous	61	40.9	32	36.8	29	46.8	
Type of drugs							
Fentanyl ^a^	131	31.0	80	49.4	51	19.6	< .0001
Morphine ^a^	11	2.6	3	1.9	8	3.1	0.5424
Midazolam^a^	38	9.0	15	9.3	23	8.9	0.8854
Paracetamol	14	3.3	9	5.6	5	1.9	0.0427
Neuromuscular blocker	7	1.7	3	1.9	4	1.5	1.0000

^a^continous and/or bolus administration in all three ventilation groups

### Drug withdrawal practices

Opioids and benzodiazepines were administered in 149 (35.3%) of the 422 infants - 113 on IV (86.3%), 26 (17.3%) on NIV, and 10 (7.1%) on SV groups - and they were weaned off the medication gradually in 59 (52.2%), 13 (50.0%) and 4 (40%) cases, respectively (*p* = 0.6782). A drug withdrawal scale was used in 38 cases (26.2%) overall - 33 on IV (29.2%), 4 on NIV (10.4%), and 1 on SV (10.0%) (*p* = 0.2349); and withdrawal was treated or prevented in 5 (4.4%), 1 (3.8%), and 0 cases, respectively. Drug withdrawal was diagnosed in 4 (3.6%) infants, who were monitored and treated with various drugs, such as methadone, morphine, phenobarbital and benzodiazepines (diazepam, midazolam, lorazepam).

### Non-pharmacological interventions

We also documented any concomitant use of sweet solutions as an adjuvant pain control measure. Almost one in two (*n* = 227 [53,8%]) of the infants admitted to an NICU received sweet solutions (more often sucrose than glucose), with no difference between the three ventilation groups: IV *n* = 69 (52.6%), NIV *n* = 88 (58.7%), SV *n* = 70 (49.6%), (*p* = 0.2900).

### Pain assessment

**Fig. 3:**
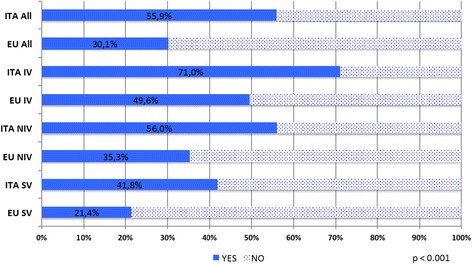
Pain assessment in Italy and all participating countries in Europe. EU include: Austria, Belgium, Cyprus, Estonia, Finland, France, Germany, Greece, Lithuania, Malta, Netherlands, Norway, Poland, Portugal, Spain, Sweden, United Kingdom. ITA = pain assessment in Italian NICU admissions, PA_EU = pain assessment in other European NICU admissions, IV = pain assessment in invasive ventilation group, NIV = pain assessment in non-invasive ventilation group, SR = pain assessment in spontaneous ventilation group. Chi-square test *p* < 0.001

Bedside PA using pain scales was recorded for 285 (67.5%) neonates in all groups- 112 on IV (85.5%), 101 on NIV (67.3%), and 72 (51.1%) on SV groups (*p* < .0001). They were used more often in Italy than in the rest of European countries (*p* < .0001), though some other countries reported assessing larger proportions of infants (88% in France, 80% in the Netherlands). Figure 3 shows the frequency of PA performed in Italy vis-à-vis the rest of Europe for the three types of ventilation.

Regarding algometric tools, prolonged pain scales were used more often than acute pain scales. The EDIN scale was used in 78.6% of cases, and the Comfort-Comfort “behavior” scale only rarely (4.6%), the PIPP in 10.9% of cases, the CRIES in 7.7%, the indirect VAS in 7.4%, and the NIPS in 5.3%. The variability between NICUs concerning this parameter was high, and 20 of the 28 (71.4%) units performed PA. The mean ± SD number of PA per infant per day was 2.99 ± 5.04.

### Factors associated with use of analgesia/sedation and pain assessment

Judging from our logistic regression model, the factors promoting the use of some form of A/S included: more severe illness, as defined by higher CRIB scores (CRIB, OR 1.46, 95%CI 1.20-1.77, *p* = 0.0002), invasive ventilation (IV vs SV OR 62.59, 95% CI 22.01-177.97, NIV vs SV OR 2.60, 95% CI 1.02-6.61*p* < 0.0001), bedside pain assessment with a scale (OR 5.38, 95%CI 1.69-17.16), and a number of bed below 15 (OR 3.55 95%CI 1.08-11.69) (Table 4). The factors promoting the use of PA, on the other hand, were: gestational age below 33 weeks (OR 8.13, 95% CI 2.64-25.03, *p* = 0.0003), invasive ventilation (IV vs SV OR 163.03, 95% CI 19.35- + ∞), NIV vs SV OR 6.06, 95% CI 2.17-16.95, *p* < 0.0001) and presence of a pain team (OR 114.35, 95% CI 1.04 - + ∞) (Table 5).

**Table 4:** Univariate and multivariate logistic regression models for factors promoting the use of analgesia sedation

Predictors of analgesia/sedation	Univariate		Multivariate	
	*p*-value	OR (95% CI)	*p*-value	OR (95% CI)
Gestational age (weeks) ≤32	<.0001	3.54 (2.15–5.83)		
CRIB score	<.0001	2.03 (1.66–2.48)	0.0002	1.46 (1.20–1.77)
Asphyxia, APGAR <5 at 5’	<.0001	33.17 (6.17–178.29)		
Sex, female	0.2999	0.78 (0.49–1.25)		
Born in same hospital as NICU	0.0307	0.497 (0.26–0.96)		
Type of ventilation				
Invasive vs Spontaneous	<.0001	117.16 (43.88–312.82)	<.0001	62.59 (22.01–177.97)
Noninvasive vs Spontaneous		2.61 (1.14–5.98)		2.60 (1.02–6.61)
Use of pain scales	<.0001	19.04 (5.92–61.19)	0.0046	5.38 (1.69–17.16)
Pain specialist physician	0.2891	0.50 (0.14 – 1.81)		
Pain specialist nurse	0.1111	0.41 (0.14–1.23)		
Pain management team	0.4972	1.42 (0.52–3.91)		
Local guidelines for pain assessment	0.8953	1.10 (0.27–4.55)		
NICU beds (≤ 15 beds vs >15 beds)	0.0089	3.68 (1.39–9.77)	0.0372	3.55 (1.08–11.69)
Number of NICU admission	0.0677	0.99 (0.98–1.00)

**Table 5:** Univariate and multivariate logistic regression models for factors promoting the use of pain assessments

Predictors of pain assessment	Univariate	Multivariate		
	*p*-value	OR (95% CI)	*p*-value	OR (95% CI)
Gestational age (weeks) ≤32	<.0001	14.05 (5.39–36.63)	0.0003	8.13 (2.64–25.03)
CRIB score	0.0005	1.77 (1.29-2.43)		
APGAR <5 at 5’	0.0381	12.14 (1.15–128.49)		
Sex, female	0.7335	0.90 (0.50–1.63)		
Born in same hospital as NICU	0.1091	0.47 (0.18–1.19)		
Type of ventilation	<.0001		<.0001	
Invasive vs Spontaneous		253.33 (33.88- + ∞)		163.03 (19.35- + ∞)
Noninvasive vs Spontaneous		10.86 (4.01–29.40)		6.06 (2.17–16.95)
Pain specialist physician	0.3706	4.51 (0.17–123.10)		
Pain specialist nurse	0.5546	2.36 (0.14–41.10)		
Pain management team	0.0342	16.51 (1.23–221.03)	0.0481	114.35 (1.04 - + ∞)
NICU beds (≤ 15 beds vs >15 beds)	0.8749	0.81 (0.06–11.61)		
Number of NICU admission	0.1075	0.95 (0.90–1.01)		

## Discussion

In this study, we analyzed the bedside pain control practices adopted at Italian NICUs, based on data collected for the prospective, multicenter EUROPAIN study. The use of A/S in the neonatal period is poorly documented and few surveys have reported on the type and frequency of the drugs used or on any pain assessments performed [3, 18–20]. Thus, this is the first Italian study that documents actual (not declarative) NICU practices regarding sedation and analgesia

We found that 35.3% of all infants admitted to the NICU in Italy received some form of A/S in the first 28 days of life. This applied mainly to patients on invasive ventilation (86.3% of cases, as opposed to 17.3% of those on NIV, and 7.1% of those on SV). Strong analgesics were given, at least once, to 83.2% of infants on IV, and sedatives to one in three of them. There was a marked variability in the use and mode of administration of A/S medication from one NICU to another. Pain assessment was documented for most NICU admissions (67.5%), but here again the situation varied considerably – and pain is clearly still far from being widely considered as the fifth vital sign.

In a previous study of ours on this topic, pharmacological interventions were reportedly used routinely in newborn on invasive and noninvasive mechanical ventilation at 87% and 26% of Italian NICUs, respectively [20]. The present report certainly better reflects day-to-day clinical practice, and our findings for Italy are consistent with the picture seen elsewhere in Europe. Some differences emerge in the types of drug used, however. In Italy, fentanyl is preferred to morphine (used in 78.6% and 8.4%, respectively, of infants on IV). The pharmacokinetics of fentanyl make it an effective analgesic because of the faster onset and relatively short duration of its action, and it has more limited hypotensive effects than morphine, especially in more preterm infants.

Similar patterns of opioid use were reported in Spain (in 79.1% of infants on IV) and Germany (91.2%), while morphine was the drug of choise in the UK (91.6%), Cyprus (100%), Lithuania (95.5%) and the Netherlands (70.5%). Sufentanil was only used in France (52.5%) and Poland (38.0%) [21], although the data available on its use in the newborn is still limited [23]. None of the infants enrolled in this study were treated with remifentanyl or alfentanyl at Italian NICUs, as in other european countries [3]. When a stronger sedative effect is needed, one in three newborn infants is given midazolam (mostly in association with opioids), despite the lack of clinical evidence to support its use in this setting [24, 25]. In particular the use of midazolam in preterm infants is not recommended as there are some concerns of its impact on neurological outcome [26]. Neuromuscular blockers are rarely used in Italy (in 6.2% of infants on IV, regardless of gestational age), unlike other European countries (UK 59.5%, Norway 46.7%, Belgium 41.2%, Finland 35.4%). There is evidence to support the use of neuromuscular blockers to facilitate and expedite tracheal intubation [27, 28], while their use during the mechanical ventilation of the newborn is less well documented [29].

Regarding the mode of A/S administration, we documented NICU staff efforts to modulate the treatment, usually by combining continuous infusions with boluses. Only one in four neonates was treated with boluses alone, as recommended in the latest Italian guidelines, [26] especially for the less sick, more preterm infants, in an attempt to reduce their cumulative dose of opioids with no loss of optimal pain control [12]. This is because of reports on the use of strong analgesics early in life being correlated with potential neurodevelopmental deficits in later infancy and childhood. Preclinical studies have suggested that opioids have negative effects on brain maturation due to their interference in the processes of neural cell differentiation, proliferation and apoptosis [30]. In a rat model, early exposure to opiates resulted in lower levels of brain-derived neurotrophic factor, a marker of synaptic plasticity and a modulator of cognitive function [30]. The opiate most often studied is morphine, which has a debated impact on the neurodevelopment of human newborn. Follow-up at 5–7 years of a small subgroup of the NEOPAIN trial showed no differences in overall intelligence quotient, but children exposed to morphine were impaired on visual analysis and short-term memory, and had more social problems than controls given placebo [13, 14]. Subsequent assessments at age 8–9 years old did not confirm these impairments, however [31]. More recently, Zwicker et al. conducted a prospective cohort study, and reported that a 10-fold increase in morphine exposure was associated with a 5.5% reduction in cerebellar volume, which correlated with worse motor and cognitive outcomes [32]. There may have been a sizable enrollment bias in their study, however.

On the other hand, several studies have confirmed the long-term neurodevelopmental impact of repetitive neonatal pain. An altered myelination at school age was recently found to be associated with a larger number of invasive procedures during hospitalization for very preterm birth in children with no severe brain injury or neurosensory impairments; a lower intelligence quotient was also associated with a larger number of invasive procedures and an altered brain microstructure [5].

Continuing uncertainty regarding how to strike the right balance between such potential outcomes should prompt a more rational and customized clinical approach to the management of pain in newborn, as Diendl et al. recently explained [33].

Analgesia and sedation in newborn infants is justified for several reasons, to control the clinical instability caused by pain and stress, unsynchronized breathing and suboptimal ventilation [34], and to prevent pain sensitization, and the long-term effects of pain on the developing brain [15, 35].

A Cochrane review concluded that ongoing analgesia with opioids during mechanical ventilation in preterm and term newborn is effective in reducing pain and stress scores, and does not prolong ventilation, alter mortality rates or subsequent intelligence, motor function, or behavior, whereas there is not enough evidence to support the *routine* use of opioid therapy for ventilated newborn infants [36]. In other words, a judicious use should be made of strong analgesics and sedatives based on regular clinical and algometric assessments – as recommended in the Italian national guidelines. The goal is to use the minimum effective dose for the prevention of behavioral and physiological derangement due to uncontrolled pain and stress, thus reducing the cumulative dose required and its potential side efffects. This is hugely important because the more premature infants are, the more they are exposed to A/S, as highlighted in the present study.

Consistently with previous studies, our logistic regression analyses revealed independent associations between A/S and type of ventilation, the use of PA [37], and illness severity [38].

The use of non-pharmacological interventions was not thoroughly documented in this study, but - in clinical practice – several methods are used in synergy with strong analgesics and sedatives to better control pain and stress, possibly sparing the infant the effects of opioids. We only documented the use of sweets solutions, administered to half of the infants in our sample, which can be helpful when administered during skin-pricking procedures to mitigate pain and thus reduce the need for additional A/S, in the more preterm infants at least.

Although the majority of Italian NICUs (82.1%) reported *routinely* conducting pain assessments, only 67% of all newborn infants admitted had a PA (85.0% of those on invasive ventilation). In the present survey, 71.4% of NICUs performed PA, and this represents an improvement over the 2012 survey, when only one in three NICUs reported doing so. Factors associated with the use of PA were a lower gestational age, invasive ventilation and the presence of a pain team. The Italian national guidelines recommend, however, routine pain assessment in all infants at least three times a day (during every nursing shift), and the outcome should be documented in the patient’s medical records, as also required by a national law (Lg N. 38/2010 on access to palliative care and pain therapy). This is an aspect of neonatal care that still needs to be improved. The proper use of PA could make pain treatments more appropriate, and needs to be encouraged. Pain assessments were based mainly on the use of tools for measuring prolonged pain (EDIN, Comfort, …) - in 78.6% of cases - whereas acute pain scales (PIPP, NIPS,CRIES) were used in only one in three cases. In clinical practice in the NICU setting, a validated scale for prolonged pain is probably more appropiate.

In interpreting our results it is important to bear in mind some limitations of our survey. First, although 28 of the 70 invited NICUs participated, most NICUs represented the northern part of Italy, but not the south of the country, thus giving us only a partial picture of NICU practices nationwide. Second, we recorded neither the dosage of the medication administered, nor all the non-pharmacological interventions that might be used (apart from sweet solutions), so we learned little about any integrated approaches to pain and stress adopted at Italian NICUs (as recommended in the national guidelines). We can also draw no conclusions on the appropriateness of the pain control measures adopted from our survey. Unfortunately, using a more complex questionnaire in an effort to collect more data would have negatively affected compliance and given rise to issues with incomplete data.

## Conclusion

We documented a generally widespred use of A/S and PA in Italy, albeit with a marked diversity between different NICUs and for infants on different types of ventilation. The recently-published national guidelines provide more advice on a judicious and appropriate use of A/S, along with recommendations for routine pain assessment and a more customized, safe and effective pain control in such a vulnerable patient population. Our data will allow us to monitor the evolution of A/S and PA practices following the latest national guidelines.

## Additional file

Additional file 1:
**Table S1.** Demographics of the study population < 33 weeks gestational age. (DOC 55 kb)

## Abbreviations

A/S: Analgesia/Sedation
AAP: American Academy of Pediatrics
CI: Continous infusion
CRIB: Clinical Risk Index for Babies
IV: Invasive ventilationNICU, Neonatal Intensive Care Unit
NIV: Noninvasive ventilation
PA: Pain Assessment
SV: Spontaneous ventilation
