# Supplementary material for: Sedation and analgesia practices at Italian neonatal intensive care units: results from the EUROPAIN study
Source: Ital J Pediatr. 2017 Mar 7;43:26. doi: 10.1186/s13052-017-0343-2 (PMC5341165; doi:10.1186/s13052-017-0343-2)
Supplement: Additional file 1: — Table S1. Demographics of the study population < 33 weeks gestational age. (DOC 55 kb) [file 13052_2017_343_MOESM1_ESM.doc]

Additional file 1: Table S1. Demographics of the study population < 33 weeks gestational age.

|  | Total  (n=162) | Tracheal ventilation  (n=78) | Non invasive ventilation  (n=70) | Spontaneous ventilation  (n=14) | p |
| --- | --- | --- | --- | --- | --- |
| Gestational age (weeks)  mean (SD)  median (IQR) | 29.2 (2.7)  29.9 (25.8-31.7) | 28.0 (2.6)  27.7  (25.8-29.9) | 30.2 (2.0)  30.8  (28.7-31.9) | 31.0 (2.9)  32.1  (31.3-32.7) | <.0001 |
| Gestational age (weeks)  24-29 N (%)  30-32 N (%) | 86 (53.1)  76 (46.9) | 59 (75.6)  19 (24.4) | 25 (35.7)  45 (64.3) | 2 (14.3)  12 (85.7) | <.0001 |
| Birth weight (g)  mean (SD)  median (IQR) | 1247 (456)  1180  (888-1614) | 1085 (438)  929  (779-1383) | 1374 (412)  1344  (1015-1755) | 1518 (464)  1555  (1160-1905) | <.0001 |
| Sex  Male N (%) | 87 (53.7) | 47 (60.3) | 35 (50.0) | 5 (35.7) | 0.169 |
| Born in same hospital as NICU N (%) | 134 (82.7) | 65 (83.3) | 60 (85.7) | 9 (64.3) | 0.151 |
| Type of delivery  Caesarean N (%) | 129 (79.6) | 62 (79.5) | 58 (82.9) | 9 (64.3) | 0.289 |
| Age ad admission (h)  mean (SD)  median (IQR) | 81,2 (313,1)  0,3 (0-0,7) | 55,3 (226,0)  0,3 (0-0,6) | 67,5 (283,2)  0,2 (0-0,6) | 294,2 (657,2)  0,6 (0-203,3) | 0.027 |
| CRIB score  mean (SD)  median (IQR) | 1 (3)  0 (0-1) | 4 (3)  2 (1-7) | 1 (2)  1 (0-1) | 1 (3)  0 (0-1) | <.0001 |
| APGAR score at 5'  mean (SD)  median (IQR) | 8 (2)  8 (7-9) | 7 (2)  7 (6-8) | 8 (1)  8 (8-9) | 9 (1)  10 (9-10) | <.0001 |
| Intubated at admission N (%) | 56 (34.6) | 56 (71.8) | NA | NA | NA |
| Duration of TV (hours)  mean (SD)  median (IQR) | NA | 157.3 (189.7)  85.0  (22.3-243.5) | NA | NA | NA |
| Duration of NIV (hours)  mean (SD)  median (IQR) | NA | N. 69  268.5 (207.9)  242.5  (77.5-468.8) | N. 70  122.2 (154.4)  56.5  (23.2-165.4) | NA | NA |
| Status at discharge  Dead N (%) | 10 (6.2) | 10 (12.8) | 0 (0.0) | 0 (0.0) | 0.003 |
| Days of participation  mean (SD)  median (IQR) | 22 (8)  28 (16-28) | 24 (8)  28 (33-28) | 21 (9)  25 (13-28) | 17 (8)  17 (12-24) | 0.013 |
| Analgesia and sedation | 75 (46.3%) | 66 (84.6%) | 19 (17.1%) | 2 (14.3) | <.0001 |
| Drug withdrawal | 3 (3.5) | 3 (4.5) | 00 (0.0) | 00 (0.0) | 0.624 |
| Any Pain assessment | 142 ( 87.7) | 70 (89.7) | 60 (85.7) | 12 (85.7) | 0.738 |

Values were missing for some variables. NA= Not applicable. CRIB= Clinical Risk Index for Babies
